# Transcriptome analysis-identified long noncoding RNA CRNDE in maintaining endothelial cell proliferation, migration, and tube formation

**DOI:** 10.1038/s41598-019-56030-9

**Published:** 2019-12-20

**Authors:** Matthew Moran, Xiao Cheng, Mohamed Sham Shihabudeen Haider Ali, Nishikant Wase, Nghi Nguyen, Weilong Yang, Chi Zhang, Concetta DiRusso, Xinghui Sun

**Affiliations:** 1 Department of Biochemistry, University of Nebraska - Lincoln, Lincoln, Nebraska 68588 USA; 2 Center for Plant Science Innovation, School of Biological Sciences, University of Nebraska - Lincoln, Lincoln, Nebraska 68588 USA; 3 Nebraska Center for Integrated Biomolecular Communication, University of Nebraska - Lincoln, Lincoln, Nebraska 68588 USA; 4 Nebraska Center for the Prevention of Obesity Diseases through Dietary Molecules, University of Nebraska - Lincoln, Lincoln, Nebraska 68588 USA

**Keywords:** Cell biology, Long non-coding RNAs

## Abstract

Obesity is a leading risk factor for type-2 diabetes. Diabetes often leads to the dysregulation of angiogenesis, although the mechanism is not fully understood. Previously, long noncoding RNAs (lncRNAs) have been found to modulate angiogenesis. In this study, we asked how the expression levels of lncRNAs change in endothelial cells in response to excessive palmitic acid treatment, an obesity-like condition. Bioinformatics analysis revealed that 305 protein-coding transcripts were upregulated and 70 were downregulated, while 64 lncRNAs were upregulated and 46 were downregulated. Gene ontology and pathway analysis identified endoplasmic reticulum stress, HIF-1 signaling, and Toll-like receptor signaling as enriched after palmitic acid treatment. Moreover, we newly report enrichment of AGE-RAGE signaling pathway in diabetic complications, IL-17 signaling, and cysteine and methionine metabolism by palmitic acid. One lncRNA, Colorectal Neoplasia Differentially Expressed (CRNDE), was selected for further investigation. Palmitic acid induces CRNDE expression by 1.9-fold. We observed that CRNDE knockdown decreases endothelial cell proliferation, migration, and capillary tube formation. These decreases are synergistic under palmitic acid stress. These data demonstrated that lncRNA CRNDE is a regulator of endothelial cell proliferation, migration, and tube formation in response to palmitic acid, and a potential target for therapies treating the complications of obesity-induced diabetes.

## Introduction

Diabetes and obesity have become major health problems throughout the world. As of 2017, approximately 24,710,000 people in the United States had diabetes, with an estimated economic impact of $327 billion^1^. One of the major processes dysregulated in diabetes is angiogenesis, i.e., the formation of new capillaries from existing ones^2^. One of the major components of angiogenesis is endothelial cells. Endothelial cells line the inner walls of the blood vessels and form a barrier between the blood and tissues. Angiogenesis begins when endothelial cells receive pro-angiogenic signals from hypoxic or injured tissues^3^. In response, endothelial cells migrate through the tissue following the gradient of angiogenic signals from both sides, proliferating to create a new vessel, until they fuse and form a new capillary^2^. A complete picture, however, has yet to be developed of how endothelial cells function and how they are regulated in obesity that lead to endothelial dysfunction, especially regarding the role of lncRNAs.

LncRNAs are defined as RNA molecules that are longer than 200 nucleotides and do not code for protein. They have been shown to affect numerous processes, such as development and tumorogenesis^4^. LncRNAs, such as metastasis associated lung adenocarcinoma transcript 1 (MALAT1), MANTIS, myocardial infarction associated transcript (MIAT) and antisense non-coding RNA in the INK4 locus (ANRIL)^5–8^, have been found to regulate angiogenesis by adopting a variety of roles. Knockdown of the lncRNA MALAT1 causes dysregulated proliferation of endothelial cells and inhibition of recovery from hindlimb ischemia^6^. MANTIS is a lncRNA found to be required for transcription of angiogenesis genes, such as SOX18 and SMAD66^5^. MIAT promotes angiogenesis by acting as a competing endogenous RNA to miR-150-5p^8^, and ANRIL inhibits angiogenesis through binding to the Polycomb Repressor Complex 2^7^.

Obesity is a leading risk factor for type 2 diabetes, and it is associated with increasing levels of circulating free fatty acids, especially palmitic acid^9,10^. Thus, palmitic acid is often used as an inducer *in vitro* to mimic obesogenic conditions present *in vivo*. Palmitic acid can cause endothelial dysfunction by interfering with normal signaling pathways. For example, it impairs insulin signaling, eNOS activity, and nitric oxide production by inducing the activation of IκB-kinase β^11^ or promoting PTEN activity^12^. It induces superoxide production through NADPH oxidase^13^, which is mediated by Toll-like receptor 4^14^. Recently, it was reported that palmitic acid treatment can activate the Stimulator of Interferon Genes (STING) by inducing mitochondrial damage and leakage of mitochondrial DNA into the cytosol^15^. Despite these exciting studies, the transcriptome regulated by palmitic acid in endothelial cells and the role of lncRNAs in regulating endothelial response to palmitic acid are not completely understood. In this study, an understanding of the transcriptome was acquired through RNA-seq and a highly expressed lncRNA, CRNDE, was selected for further study to determine its effects on angiogenesis. It is observed that palmitic acid upregulates genes involved in stress response and reduces angiogenic function. Also, knockdown of the lncRNA CRNDE inhibits angiogenic potential in endothelial cells.

## Results

### Analysis of HUVEC transcriptome

**Figure 1:**
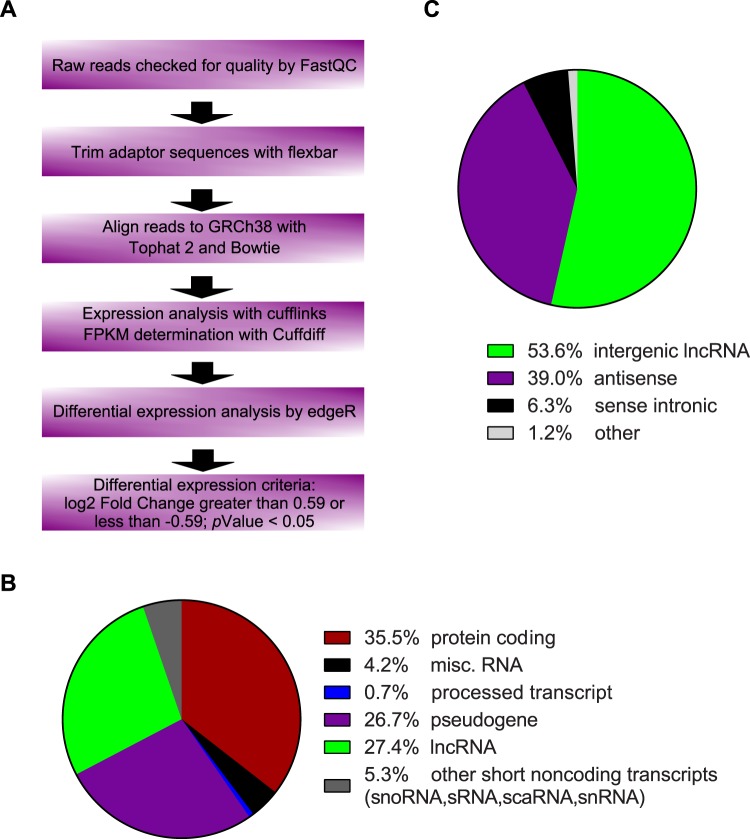
Distribution of RNA-seq data. (**A)** Analysis workflow for RNA-seq data. (**B**) Percentage of the different types of RNA identified by RNA-seq. (**C**) Percentages of the different types of lncRNA identified in (**B**).

**Figure 2:**
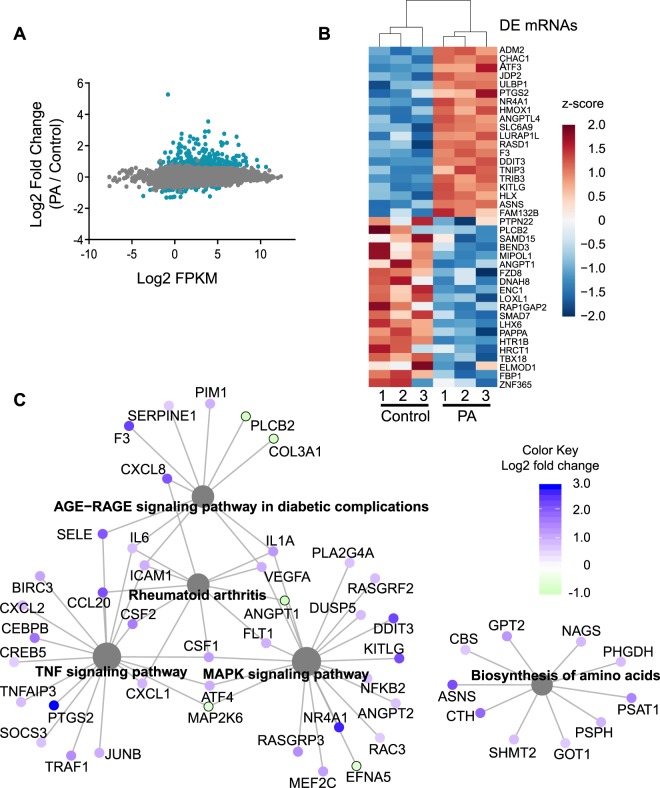
Differentially expressed mRNAs. (**A**) Expression plot of identified mRNAs. Expression is determined by average FPKM. Blue are those mRNAs significantly differentially expressed. (**B**) Heatmap of the top 20 upregulated and top 20 downregulated mRNAs. Data was plotted as z-score of the log2 fold change. (**C**) Highly enriched KEGG pathways from differentially expressed genes. Protein coding genes are identified by gene symbols.

**Figure 3:**
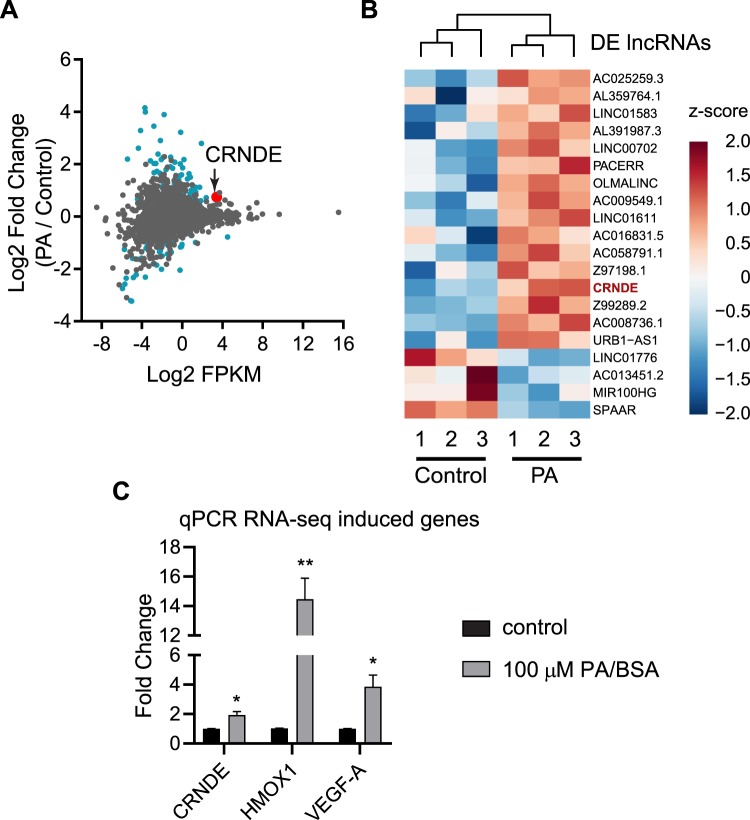
Differentially expressed lncRNAs. (**A**) Expression plot of identified lncRNAs. Expression is determined by average FPKM. Blue and red are those lncRNAs significantly differentially expressed; red is CRNDE. (**B**) Heatmap of the top 20 lncRNAs by expression that are differentially expressed. Data was plotted and ranked as z-score of the log2 fold change. (**C**) Effect of palmitic acid on gene expression. HUVECs were treated with palmitic acid or vehicle control for 12 hours. The expression of CRNDE, HMOX1, and VEGF-A was analyzed by RT-qPCR, *p < 0.05, **p < 0.001.

To determine the effect that palmitic acid has on endothelial transcriptome, RNA-seq was performed. Human umbilical vein endothelial cells (HUVECs) were treated with 100 μM palmitic acid and RNA sequencing was performed. After alignment and differential expression analysis, according to the process outlined in Fig. 1A, principal component analysis of the clusters was performed, and the palmitic acid treatment and control (fatty acid-free BSA) clustered as expected (Supplementary Fig. 1A). 35.5% of transcripts analyzed are mRNA and 27.4% are lncRNA (Fig. 1B). There were between 60 million and 80 million reads mapped for the samples (Supplementary Table 1). An mRNA was considered as expressed if the average counts per million (CPM) in one condition (control or PA) was greater than 1. Of the 27.4% mapped as lncRNA, 53.6% was intergenic lncRNA, which was analyzed further in this study (Fig. 1C). LncRNA was considered expressed if the CPM in one condition was greater than 0.1 to include lncRNAs expressed at low levels in the analysis as lncRNA expression levels are 10-fold less abundant than mRNAs^16^. All mRNAs or lncRNAs that were not found to be expressed were eliminated from the analysis. mRNAs and lncRNAs were considered as significantly differentially expressed if the fold change of palmitic acid versus control was greater than 1.5 or less than 0.66 and the p-value was less than 0.05. Analysis based on mapping to individual chromosomes found that the highest number of differentially expressed mRNAs and lncRNAs were on chromosome 1 (Supplementary Figs. 1B and 2A). Analysis of the expression levels of the mRNA found that the transcripts which were differentially expressed had a wide range of expression (Fig. 2A). Heatmap analysis of the differentially expressed mRNA showed 305 protein-coding transcripts upregulated after palmitic acid treatment compared to control, and 70 protein-coding transcripts downregulated (Supplementary Fig. 1C). The top 20 upregulated and 20 downregulated mRNAs were shown in Fig. 2B. We found that the top enriched Kyoto Encyclopedia of Genes and Genomes (KEGG) pathways in differentially expressed genes were linked to the tumor necrosis factor-α (TNF-α), MAPK, NF-κB, PPAR, HIF-1 signaling pathways and insulin resistance (Fig. 2C, Table 1). In addition, we discovered that AGE-RAGE signaling pathway in diabetic complications and IL-17 signaling were enriched, which have not been reported. Gene Ontology (GO) analysis by Gene Set Enrichment Analysis of genes induced by palmitic acid showed a number of enriched GO terms (Table 2). Palmitic acid induces endoplasmic reticulum (ER) stress^17,18^, and this was also shown by being the most enriched GO terms in our RNA-seq (Table 2). Given this, we examined the effect of CRNDE knockdown on ER stress response genes. CRNDE knockdown reduces the ER stress response (Supplementary Fig. 1D), as measured by the ER stress response genes DNA damage inducible transcript 3 (DDIT3), PRKR-like endoplasmic reticulum kinase (PERK, also known as eukaryotic initiation factor 2 alpha kinase 3), X-box binding protein 1 (XBP1) and activating transcription factor-6 (ATF6). These data suggest that CRNDE is involved in the upregulation of ER stress response genes by palmitic acid. Analysis of genes reduced by palmitic acid showed enrichment in GO terms such as Wnt protein binding, Wnt activated receptor activity, transforming growth factor beta binding, and transforming growth factor beta receptor binding (Table 3). Expression level analysis of differentially expressed lncRNAs, using the same fold change cutoff as mRNA, showed the same range of expression as mRNA, although some of the most highly expressed lncRNA were not significantly differentially expressed (Fig. 3A). Heatmap analysis of the significantly differentially expressed lncRNAs showed that 64 lncRNAs were upregulated and 46 downregulated (Supplementary Fig. 2B; Supplementary Table 3). CRNDE (indicated by the red dot in Fig. 3A) is one of the highest expressed lncRNAs that was also significantly induced by palmitic acid. The top 20 lncRNAs by expression that are also differentially expressed were shown in Fig. 3B. Our data shows that the expression of many protein-coding genes and lncRNAs were changed by palmitic acid in HUVECs.

**Table 1:** Enriched KEGG pathways in differentially expressed genes.

Pathway	No. of genes	FDR
TNF signaling pathway	18/110	2.13 × 10^−8^
Rheumatoid arthritis	11/91	8.02 × 10^−4^
Biosynthesis of amino acids	10/75	8.02 × 10^−4^
AGE-RAGE signaling pathway in diabetic complications	11/100	1.41 × 10^−3^
MAPK signaling pathway	19/295	3.70 × 10^−3^
NF-kappa B signaling pathway	10/100	4.69 × 10^−3^
Transcriptional misregulation in cancer	14/186	4.69 × 10^−3^
Glycine, serine and threonine metabolism	6/40	9.15 × 10^−3^
IL-17 signaling pathway	9/93	9.15 × 10^−3^
PPAR signaling pathway	8/76	9.84 × 10^−3^
Fluid shear stress and atherosclerosis	11/139	1.00 × 10^−2^
HIF-1 signaling pathway	9/100	1.17 × 10^−2^
Cysteine and methionine metabolism	6/49	1.67 × 10^−2^
Insulin resistance	9/108	1.67 × 10^−2^
One carbon pool by folate	4/20	1.67 × 10^−2^
Ras signaling pathway	14/232	1.90 × 10^−2^
Rap1 signaling pathway	13/208	1.92 × 10^−2^
Kaposi sarcoma-associated herpesvirus infection	12/186	2.10 × 10^−2^
Amoebiasis	8/95	2.26 × 10^−2^
Ferroptosis	5/40	2.75 × 10^−2^

No. of genes is reported as the number of genes in the pathway identified as differentially expressed in this data set versus the total number of genes in the pathway.

**Table 2:** Top 5 Gene Ontology terms from each category enriched in genes upregulated by palmitic acid.

Category	Name	No. of Genes	FDR
Biological Process	Intrinsic Apoptotic Signaling Pathway in Response to Endoplasmic Reticulum Stress	30/32	1.03 × 10^−4^
	Response to Endoplasmic Reticulum Stress	196/233	7.81 × 10^−4^
	Decidualization	14/21	1.91 × 10^−3^
	Cellular Response to Topologically Incorrect Protein	99/122	1.98 × 10^−3^
	Positive Regulation of Transcription From RNA Polymerase II Promoter umn Response to Stress	18/23	3.03 × 10^−3^
Cellular Component	Nuclear Nucleosome	30/41	5.20 × 10^−3^
	DNA Packaging Complex	79/108	7.01 × 10^−3^
	Anchored Component of Membrane	38/152	6.65 × 10^−2^
	Nuclear Euchromatin	20/24	1.21 × 10^−1^
	Pre Autophagosomal Structure	25/31	1.32 × 10^−1^
Metabolic Function	Amino Acid Transmembrane Transporter Activity	39/79	2.85 × 10^−3^
	Hormone Activity	16/119	6.59 × 10^−3^
	L Amino Acid Transmembrane Transporter Activity	31/54	1.01 × 10^−2^
	Anion Cation Symporter Activity	17/53	1.85 × 10^−2^
	LRR Domain Binding	14/17	2.01 × 10^−2^

No. of genes is reported as the number of genes in the term identified as differentially expressed in this data set versus the total number of genes in the term.

**Table 3:** Top 5 Gene Ontology terms from each category enriched in genes downregulated by palmitic acid.

Category	Name	No. of Genes	FDR
Biological Process	Spliceosomal snRNP Assembly	33/38	8.47 × 10^−2^
	Maturation of SSU rRNA	39/42	1.23 × 10^−1^
	Nucleobase Metabolic Process	30/39	1.33 × 10^−1^
	Nucleobase Biosynthetic Process	18/18	1.49 × 10^−1^
	Regulation of Heart Morphogenesis	16/29	1.58 × 10^−1^
Cellular Component	Small Subunit Processome	30/33	8.07 × 10^−2^
	Preribosome	54/61	9.26 × 10^−2^
	Small Nucleolar Ribonucleoprotein Complex	18/20	9.46 × 10^−2^
	SMN Sm Protein Complex	17/17	1.12 × 10^−1^
	Nucleolar Part	56/62	1.52 × 10^−1^
Metabolic Function	Wnt Protein Binding	16/31	2.06 × 10^−1^
	Nucleosomal DNA Binding	28/30	2.09 × 10^−1^
	Wnt Activated Receptor Activity	13/22	2.23 × 10^−1^
	Transforming Growth Factor Beta Binding	15/16	2.32 × 10^−1^
	Transforming Growth Factor Beta Receptor Binding	27/50	2.39 × 10^−1^

No. of genes is reported as the number of genes in the term identified as differentially expressed in this data set versus the total number of genes in the term.

### LncRNA CRNDE expression is induced by palmitic acid in HUVECs

The lncRNA CRNDE was chosen for further study, since it is highly expressed in HUVECs and its expression was significantly induced by palmitic acid (Fig. 3B). CPMs for CRNDE are shown in Supplementary Table 2. CRNDE upregulation was confirmed by qPCR after HUVECs were treated with 100 µM palmitic acid. After 12 hours, CRNDE was significantly upregulated by 1.9-fold (Fig. 3C). HMOX1 and VEGF-A, which are well known genes involved in angiogenesis^19,20^, were also analyzed to compare the relative expression of CRNDE with them (Fig. 3C). To examine the mechanisms underlying CRNDE induction by palmitic acid in HUVECs, the proximal promoter of CRNDE was cloned into luciferase reporter (Supplementary Fig. 3). The CRNDE promoter was defined as a sequence of 1761 nucleotides surrounding the transcription start site encompassing 1718 nucleotides upstream of the *Crnde* gene and 43 nucleotides of 5′ *Crnde* gene. The luciferase activities were increased by 8.1-fold in the presence of CRNDE promoter, indicating CRNDE promoter indeed activates luciferase expression. However, the luciferase activities driven by CRNDE promoter were not further increased in HUVECs by palmitic acid treatment compared with control, suggesting a distal cis-sequence mediates CRNDE induction by palmitic acid. Given the effect of palmitic acid upregulating NF-κB signaling, it was unknown whether palmitic acid induces CRNDE expression through NF-κB signaling. HUVECs were treated with TNF-α to activate NF-κB signaling, then the expression of CRNDE expression was examined by qPCR. It turned out the CRNDE expression was not TNF-α-responsive (Supplementary Fig. 4), suggesting the induction of CRNDE by palmitic acid is not likely mediated by palmitic acid-activated NF-κB signaling.

### CRNDE knockdown inhibits angiogenic potential

**Figure 4:**
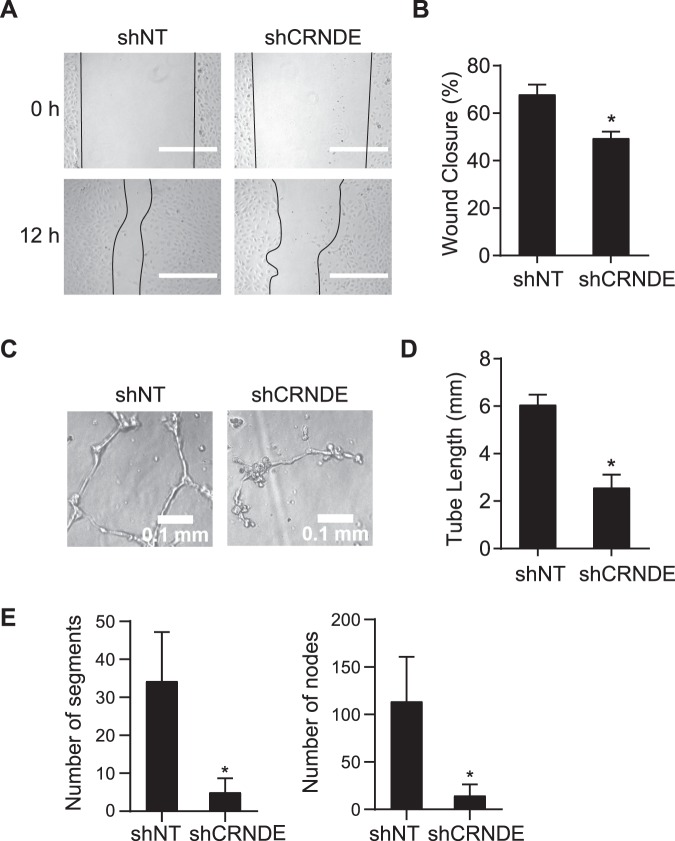
CRNDE knockdown inhibits endothelial cell proliferation and tube formation. (**A**) CRNDE knockdown reduces wound closure in HUVECs. CRNDE was treated with CRNDE shRNA (shCRNDE) or non-targeting control shRNA (shNT) for 24 h before wounding. Images were taken from fixed cells 12 h after wounding. Representative images of 3 independent experiments were shown. Scale bars are 500 µm. (**B**) Quantification of wound closure, *p = 0.027. (**C**) CRNDE knockdown reduces tube formation. HUVECs were treated as in a before being seeded into Matrigel-coated wells. Images were taken from fixed wells after 6 hours. Representative images of 3 independent experiments were shown. (**D**) Quantification of tube length in millimeters *p = 3.2 * 10^−6^. (**E**) Quantification of number of segments (*p = 9.9 * 10^−4^) and number of nodes (*p = 6.5 * 10^−4^).

**Figure 5:**
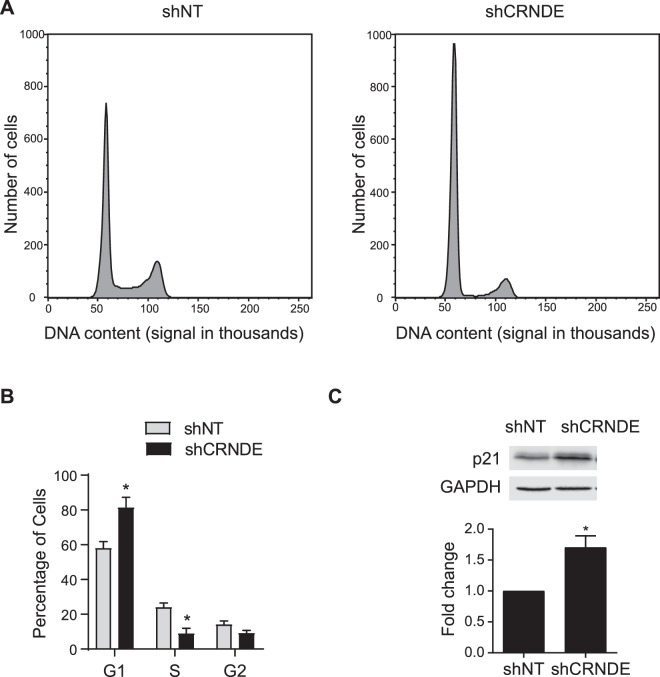
CRNDE knockdown inhibits the progression of cell cycle. (**A**) Histogram of DNA content in HUVECs after CRNDE knockdown. HUVECs were treated with shNT or shCRNDE and then stained with propidium iodide. DNA content was measured by flow cytometry at 488 nm. Representative histograms of three independent experiments were shown. (**B**) Quantification of cell cycle phase in three independent experiments, *p < 0.05. (**C**) Western blot analysis of p21 expression and quantification of three independent experiments (*p = 1.4 * 10^−3^).

To determine the effect of CRNDE on endothelial cells, functional assays were performed. Lentiviral shRNA against CRNDE (shCRNDE) was used to knock down CRNDE expression, and endothelial cell proliferation, migration, and angiogenic activity were measured. CRNDE expression was reduced by 87.5% in HUVECs transduced with lentiviral shCRNDE (Supplementary Fig. 5). CRNDE knockdown reduces the wound closure by 27.1% in the scratch-wound assay (Fig. 4A,B), indicating CRNDE knockdown inhibits cell migration. As a measure of angiogenic activity, a Matrigel® capillary-like tube formation assay^21^ was performed (Fig. 4C–E). After 6 h, total tube length, nodes and segments were reduced by 57.7%, 87.2% and 85.5%, respectively, in HUVECs transduced with lentiviral shCRNDE than in HUVECs transduced with lentiviral control shRNA, indicating that CRNDE knockdown has an inhibitory effect on the capacity of HUVECS to form capillary-like tube structures. The effects of CRNDE knockdown on cell cycle were examined by propidium iodide staining followed by flow cytometry analysis (Fig. 5A,B). The percentage of cells in S phase is 8.9% after CRNDE knockdown compared with 23.9% in control cells. In contrast, the percentage of cells in G1 phage is 81.2% compared with 57.9% in control cells. These data suggest that CRNDE knockdown inhibits cell proliferation. We examined the expression of cyclin kinase inhibitor p21 by western blot analysis and found the CRNDE knockdown increases p21 expression by 1.7-fold (Fig. 5C, Supplementary Fig. 6). These data demonstrate that CRNDE knockdown inhibits the angiogenic potential of HUVECs.

### CRNDE is involved in the response to palmitic acid

**Figure 6:**
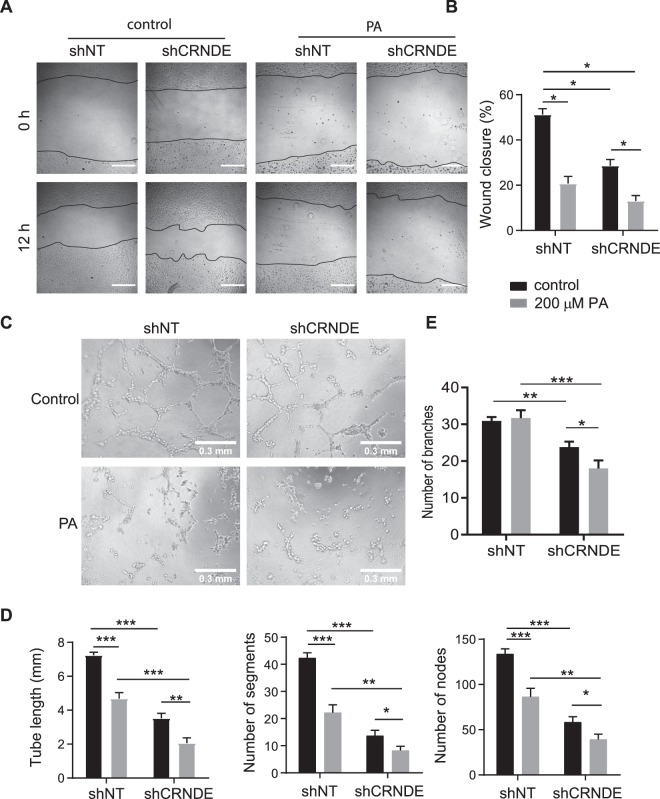
CRNDE knockdown potentiates the inhibitory effects of palmitic acid on endothelial cell migration and tube formation. (**A**) CRNDE knockdown potentiates the inhibitory effects of palmitic acid on wound closure. HUVECs were treated with shNT or shCRNDE for 24 h and then palmitic acid or vehicle control for 12 h before wounding. Images were taken from fixed cells 12 h after wounding. Representative images of 3 independent experiments were shown. Scale bars are 500 µm. (**B**) Quantification of (A) (*p < 0.001). (**C**) CRNDE knockdown potentiates the inhibitory effects of palmitic acid on tube formation. HUVECs were treated as for wound healing before being seeded into Matrigel-coated wells. Images were taken from fixed wells after 6 h. Representative images of 3 independent experiments were shown. (**D**) Quantification of tube formation in (C). Tube length in millimeters, the number of segments, and the number of nodes (*p < 0.05, **p < 0.01, ***p < 1 * 10^−4^). (**E**) Quantification of tube formation in (C). Number of branches (*p < 0.05, **p < 0.001, ***p < 1 * 10^−4^).

Having established that CRNDE is involved in angiogenesis, the role of CRNDE on angiogenesis under palmitic acid stress was determined by performing scratch and tube formation assays (Fig. 6). HUVECs were transduced with shCRNDE and treated with palmitic acid for 12 h, and then the assays were performed. In the scratch-wound assay, palmitic acid treatment reduces wound closure by 59.6% (Fig. 6A,B), which is consistent with a previous study^22^. CRNDE knockdown reduces wound closure by 44.0% in control HUVECs (Fig. 6A,B). Importantly, CRNDE knockdown and palmitic acid treatment have synergistic effects on wound closure, which was reduced by 74.6%. In the capillary-like tube formation assay, palmitic acid treatment reduces tube length by 35.4% in shNT-treated cells (Fig. 6C,D), which is consistent with what previously reported^22^. In addition, palmitic acid treatment reduces the number of segments and number of nodes by 47.7% and 35.5%, respectively (Fig. 6C,D). CRNDE knockdown reduces tube length by 51.3%, the number of segments by 67.7%, and the number of nodes by 56.3% in control HUVECs (Fig. 6C,D). Similarly, CRNDE knockdown and palmitic acid treatment have synergistic effects on capillary-like tube formation. CRNDE knockdown reduces the tube length, the number of segments, and the number of nodes by 71.6%, 80.6%, and 70.6%, respectively, in HUVECs treated with palmitic acid compared with shNT-treated cells under control condition (Fig. 6C,D). CRNDE knockdown also reduces branching by 23.4% and 43.2% under control and palmitic acid conditions, respectively; palmitic does not reduce branching in shNT-treated cells (Fig. 6E). Human aortic endothelial cells (HAECs) showed a similar trend in some respects (Supplementary Fig. 7). CRNDE knockdown reduced wound closure by 43.5% under control condition. It also reduced the number of nodes and the number of segments by 16.3% and 22% respectively (Supplementary Fig. 7). Tube length and branching were not significantly reduced. Palmitic acid reduced wound closure by 68.6% and tube length, the number of nodes, the number of segments and branching by 25.9%, 57.6%, 73.6% and 34.2% respectively in shNT-treated cells (Supplementary Fig. 7). Surprisingly, overexpressing CRNDE 87.1-fold (Supplementary Fig. 8) did not produce the opposite effect when examined by the scratch-wound and tubule formation assays (Supplementary Fig. 9). CRNDE overexpression reduces tube length, the number of segments, the number of nodes and branching by 13.3%, 21.7%, 27.2%, and 27.8% respectively (Supplementary Fig. 9). When CRNDE overexpressing HUVECs are treated with palmitic acid, wound closure is reduced by 73.1% compared to control cells (Supplementary Fig. 9). Tube length, the number of segments, the number of nodes and branching are reduced by 28.3%, 38.7%, 28.4% and 32.6% respectively (Supplementary Fig. 9). Our data imply that the induction of CRNDE by palmitic acid may counteract the effects of palmitic acid on endothelial cells.

## Discussion

In this study, changes in the transcriptome of HUVECs induced by palmitic acid were identified by RNA-seq, and the lncRNA CRNDE is shown to maintain angiogenic potential of HUVECs. CRNDE has already been identified as regulating metabolic pathways in cancer, but its role in diseases other than cancer has not been established. One way in which CRNDE is involved in regulating metabolism is in colorectal cancer, being downregulated by insulin in an AKT and MAPK dependent manner^23^. The loss-of-function studies demonstrated that CRNDE expression promotes the metabolic changes by which cancer cells switch to aerobic glycolysis^23^. CRNDE has also been identified as an oncogene, activating the Wnt signaling pathway in breast cancer by sponging miR-136^24^. Other than cancer, CRNDE is required for platelet‐derived growth factor‐BB-stimulated proliferation and migration of vascular smooth muscle cells^25^, and it inhibits the transcriptional activation of Smad3 on target genes in cardiac fibroblasts^26^. In our studies, we found that CRNDE knockdown reduces endothelial cell migration, proliferation, and capillary-like tube formation (Figs. 4–6). These data suggest that CRNDE is an important regulator of various cellular functions in different cell types and may be a potential drug target. LncRNAs can be targeted by drugs to cause degradation, just as they are knocked down *in vitro* and *in vivo*. One such mechanism is the use of chemically modified antisense oligonucleotides. One form of antisense oligonucleotides, a gapmeR, consists of two spans of modified ribonucleotides connected by an antisense DNA linker that recruits RNse H to degrade the target RNA^27,28^. GapmeRs have been successfully used to knock down lncRNA *in vitro* and *in vivo*^29–31^. One gapmeR, mipomersen, has been approved for familial hypercholesterolemia^32^.

Our transcriptome analysis by RNA-seq revealed that the expression of hundreds of transcripts including mRNAs and lncRNAs is changed by palmitic acid in HUVECs (Figs. 1–3). Gene ontology analysis also shows that palmitic acid induces endoplasmic reticulum stress (Table 2). Palmitic acid also reduces endothelial cell proliferation and migration through activating the cGAS/STING signaling pathway, a marker of mitochondrial damage^22^. Interestingly, CRNDE knockdown reduces ER stress related genes (Supplementary Fig. 1D). Endoplasmic reticulum stress and oxidative stress are linked in endothelial cells^33^, and oxidative stress can induce JAK-STAT signaling^34^. CRNDE can also induce JAK-STAT in fibroblasts after lipopolysaccharide challenge^35^. Given this, the possible connection between palmitic acid induced endoplasmic reticulum stress and CRNDE induction needs to be examined in future studies, providing a possible mechanism by which CRNDE expression is induced after palmitic acid challenge. Given that in HUVECs, CRNDE knockdown reduced endothelial cell proliferation and migration more than palmitic acid alone (Fig. 6), but CRNDE is not directly induced by palmitic acid (Supplementary Fig. 3), CRNDE may act as a compensatory mechanism to preserve some angiogenic ability after palmitic acid treatment. Palmitic acid induces other compensatory mechanisms, such as causing induction of HMOX1 (heme oxygenase 1) in neuroblastoma cells to deal with the increased oxidative stress palmitic acid causes^36^. Indeed, in this study, HMOX1 is induced by palmitic acid (Figs. 2B and 3C).

The molecular basis by which CRNDE knockdown inhibits angiogenic potential in endothelial cells remains unknown. CRNDE promotes proliferation and migration of trophoblasts by acting as a competing endogenous RNA for miR-1277, and its knockdown reduces proliferation and migration^37^. Given this, CRNDE may also affect endothelial cell proliferation and migration through effecting miRNA levels. When CRNDE is knocked down, there is a reduction in endothelial cell proliferation, and the cell cycle arrests in the G1 phase (Figs. 5 and 6). This is associated with an increase in p21 expression (Fig. 5C). This is consistent with a previous study showing that CRNDE silencing induces the expression of p21 in colorectal cancer cells^38^. Thus, increased p21 expression may mediate the effects of CRNDE knockdown on endothelial cell migration, proliferation, and capillary tube formation in HUVECs. Finally, CRNDE overexpression did not have an opposite effect to CRNDE knockdown on endothelial cell proliferation and tube formation (Supplementary Fig. 9), indicating that CRNDE is necessary but not sufficient for the induction of these processes. The CRNDE overexpression results suggest additional cofactors are required to facilitate CRNDE function. While the mechanism of CRNDE’s action in endothelial cells is unknown, CRNDE has the potential to be a novel target in the fight against the angiogenic complications of diabetes and obesity.

## Methods

All methods reported here were carried out in accordance with relevant guidelines and regulations of the University of Nebraska - Lincoln. All experimental protocols were reviewed and approved by the University of Nebraska - Lincoln Institutional Biosafety Committee (Protocol Number: IBC788). The studies in the paper did not involve human tissue samples or human subjects. There are no studies in the manuscript that require ethical approval.

### Cell culture

HUVECs from pooled donors (Cat. No. CC-2519) and Human Aortic Endothelial Cells (HAECs) (Cat. No. CC-2535) were purchased from Lonza and cultured in either EGM-2 (Lonza Cat. No. CC-3162) for RNA-seq experiments or Endothelial Cell Growth Medium (ATCC Cat. No. PCS-100-041) supplemented with 1% penicillin-streptomycin for all other experiments. Media was changed every two days during normal passaging. All cells used were between passages 3 and 8. In the tubule formation assay, Endothelial Cell Basal Medium (PromoCell Cat. No. C-22211; hereafter PromoCell EBM) was used where indicated. Cell detachment was carried out with 0.05% trypsin in EDTA (Thermo Fisher). All the experiments were done using three different lots of HUVECs.

### RNA sequencing and analysis

HUVECs were treated for 8 h with 100 µM palmitic acid (NuChuk Prep.) conjugated to fatty-acid poor BSA (EMD Millipore) in a 3 to 1 molar ratio as previously described^11^. RNA was harvested and rRNA depleted, adaptors were ligated, and the samples sent for sequencing on an Illumina HiSeq 2500. After obtaining raw reads, FastQC was used to analyze the quality of the reads. Tophat2 and bowtie^39–41^ were used to map the reads to the genome, GRCh38 (Ensembl release 82^42^), and cufflinks^43^ was used to determine transcript expression. Cuffdiff^44^ was used to generate FPKM values and edgeR^45–48^ was used for differential expression analysis. To determine differential expression, the fold change values were transformed by log2 and compared against the log2 fold change values from edgeR, that had a requisite *p*-Value (less than 0.05). Heatmaps were generated using the R pheatmap package. For Fig. 3B, gene names were taken from Ensembl Release 97, eliminating from Supplementary Table 3 those IDs which have been removed from release 97.

### KEGG pathway analysis

All significantly differentially expressed genes were used for KEGG pathway enrichment analysis. Using the biological id translator function from R clusterProfiler package^49^, Ensembl IDs were converted to NCBI gene IDs and used for KEGG pathway enrichment analysis. First KEGG pathway enrichment analysis was performed using human metabolic network as a background. P-value cut-off was set at 0.05 and Bonferroni-Hochberg adjustment was performed with minimum gene set (GS) size set to 10 and max GS to 500. Finally, KEGG pathway enrichment data was used to generate plots showing linkages of gene and enriched KEGG pathways. The edges of the nodes for reduced genes were manually highlighted with black lines.

### Gene ontology analysis

Differentially expressed mRNAs were analyzed by Genome Set Enrichment Analysis as described previously^50^. GSEA was used to perform Gene Ontology analysis. Enriched terms were reported in upregulated and downregulated genes. To determine differentially enriched gene ontology terms, a false discovery rate of 0.25 was used as a cutoff.

### CRNDE shRNA lentiviral generation and HUVEC transduction

The shRNA target sequence GTGTGATGCTTCCATAATACA was cloned into the vector pLKO.1-TRC, a gift from David Root (Addgene plasmid No. 10878) to create shCRNDE. HEK-293T cells were cultured in DMEM supplemented with 10% fetal bovine serum and penicillin/streptomycin (Thermo Fisher). Lentivirus was produced by transfecting HEK-293T cells with the CRNDE-pLKO.1 plasmid using Lipofectamine 2000 (Thermo Fisher). After 24 h transfection, media was collected at 48 h and 72 h post transfection. HUVECs were plated into 12 well plates (100,000 cells/well) and transduced with the appropriate volume of virus to knock down CRNDE. 24 hours post transduction media was changed for downstream applications.

### Reverse transcription quantitative PCR

RNA was collected from HUVECs and precipitated using TRIzol Reagent (Thermo Fisher Cat. No. 15596026) according to the manufacturer’s instructions. 1 μg of RNA was converted to cDNA using the High Capacity cDNA Reverse Transcription Kit (Thermo Fisher Cat. No. 4368813). qPCR was conducted in the CFX Connect Real Time System (BioRad) using 2x Sybr Green qPCR Master Mix (Bimake Cat. No. B21203). Data was normalized by the DeltaDeltaCt method^51^. The primers for qPCR are in Supplementary Table 4.

### Scratch assay

HUVECs or HAECs were transduced with CRNDE shRNA (shCRNDE) or a non-targeting control shRNA (shNT) for 24 h and then seeded into 2 wells of a 6-well plate per condition at 600,000 cells per well. After resting for 12 h, cells were treated with or without palmitic acid (100 or 200 μM) for 12 h. After treatment 2 wounds were produced per confluent well and images were obtained. After wound formation, wells were fixed with 2% formaldehyde and images were again taken in the same places. The wound area was measured with ImageJ and the percentage wound closure was calculated by measuring the area of the wound.

### Tube formation assay

The tubule formation assay was performed as previously described, with slight modifications^21^. HUVECs or HAECs were transduced as in the scratch assay and then seeded into one well of a 6-well plate at 300,000 cells per well. After 12 h, cells were treated with or without palmitic acid (100 or 200 μM) for 12 h. Cells were then trypsinized and neutralized with PromoCell EBM supplemented with 1% FBS. After centrifugation, HUVECs were resuspended in EBM without FBS and seeded into Matrigel (Corning Cat. No. 254234) coated wells (50 μl per well) at 15,000 cells per well. After 3–6 h, cells were fixed with 4% formaldehyde and 4–6 random images were taken per well, with duplicate wells per condition. Images were then analyzed with ImageJ using the Angiogenesis Analyzer plugin by Giles Carpentier (http://imagej.nih.gov/ij/macros/toolsets/Angiogenesis%20Analyzer.txt), and total branching length, number of nodes and number of segments were used as markers of angiogenic tube formation.

### Flow cytometry

HUVECs were treated with shCRNDE or shNT for 24 h. 24 h later, cells were fixed in 70% ethanol at 4 °C with a concentration less than 1 × 10^6^ cells per sample. After fixation, cells were treated with 200 μg/ml RNase A and stained with propidium iodide at a final concentration of 10 μg/ml. Cells were then analyzed using the DxP10 FACsort (BD). Data was analyzed using the FlowJo software (BD).

### Western blot

HUVECs were treated with shCRNDE or shNT for 24 h. 24 h later, cells were lysed in radioimmunoprecipitation buffer (25 mM Tris-HCl pH 7.6, 150 mM NaCl, 1% NP-40, 1% sodium deoxycholate, 0.1% SDS), and protein concentration determined with the Pierce BCA Protein Assay Kit (Thermo Fisher). 10 µg of protein was loaded per sample and the samples were separated by SDS-PAGE using a 12% acrylamide gel. After SDS-PAGE, the samples were transferred to a polyvinylidene fluoride membrane using the Trans Blot Turbo Transfer System (BioRad; 25 V, 1.0 A, 30 min). After blocking with 5% nonfat milk in TBST, rabbit anti-p21 (Cell Signaling Technology Cat. No. 2974) was used to probe for p21. Then the blot was probed with HRP-conjugated anti-rabbit (Cell Signaling Technology Cat. No. 7074) and the signal was detected by chemiluminescence using the LiCOR Odyssey Fc Imaging System. The blot was stripped using an SDS-glycine stripping buffer (1.5% glycine, 0.1% SDS, 1% Tween 20) and re-probed with rabbit anti-GAPDH (Cell Signaling Technology Cat. No. 2118). GAPDH was detected in the same way as p21.

### Statistical analysis

All experimental data is the average of three independent experiments, reported as mean ± standard error, unless otherwise stated. All statistical analysis was carried out using two-tailed Student’s t-test with an alpha level of 0.05. All comparisons were to the control, unless otherwise stated.

## Supplementary information

Supplementary Information

## Data Availability

The RNA-seq data generated in this manuscript is available through the Gene Expression Omnibus^52^ (GSE141126): https://www.ncbi.nlm.nih.gov/geo/query/acc.cgi?acc = GSE141126.
