# Supplementary material for: Transcriptome analysis-identified long noncoding RNA CRNDE in maintaining endothelial cell proliferation, migration, and tube formation
Source: Sci Rep. 2019 Dec 20;9:19548. doi: 10.1038/s41598-019-56030-9 (PMC6925215; doi:10.1038/s41598-019-56030-9)
Supplement: Supplementary file 1 — Supplementary Information [file 41598_2019_56030_MOESM1_ESM.pdf]

Transcriptome analysis-identified long noncoding RNA CRNDE in maintaining endothelial cell proliferation, migration, and tube formation

Matthew Moran[1], Xiao Cheng[1], Mohamed Sham Shihabudeen Haider Ali[1], Nishikant Wase[1], Nghi Nguyen[1], Weilong Yang[2], Chi Zhang[2], Concetta DiRusso[1,3], and Xinghui Sun[1,4,*]

1. Department of Biochemistry, University of Nebraska - Lincoln, Lincoln, Nebraska 68588, USA.
2. Center for Plant Science Innovation, School of Biological Sciences, University of Nebraska - Lincoln, Lincoln, Nebraska 68588, USA.
3. Nebraska Center for Integrated Biomolecular Communication, University of Nebraska - Lincoln, Lincoln, Nebraska 68588, USA.
4. Nebraska Center for the Prevention of Obesity Diseases through Dietary Molecules, University of Nebraska - Lincoln, Lincoln, Nebraska 68588, USA.

* To whom correspondence can be addressed. Tel [+1 402 472 8898]; email: xsun17@unl.edu.

Supplementary Methods

CRNDE promoter construction

The 1761 base pairs proximal to the transcription start site of CRNDE (1718 base pairs upstream of *Crnde* gene and 43 base pairs of 5' *Crnde* gene) were amplified by qPCR from human genomic DNA and considered as its promoter (Supplementary Figure 3B). This sequence was cloned into the pGL3-basic vector (Promega Cat. No. E1751) upstream of luciferase to create pGL3-CRNDE pro. Primers are: forward 5'- AATGGTACCGGGGGCTCTGCTTTTGCC-3' and reverse 5'-ACAAAGCTTAGCCAGCTTCCCGTCTCCAT-3'.

Promoter activity assay

HUVECs were seeded into a 12-well plate and transfected at 90% confluency with either control pGL3-basic vector or pGL3-CRNDE with Lipofectamine 2000 for 2 h. Transfected cells were allowed to recover for 24 h. Luciferase activity was then determined with the Luciferase Assay System kit (Promega Cat No. E4030). Briefly cells were lysed in Reporter Lysis Buffer and luminescence was measured immediately after the addition of substrate. Luminescence was normalized to µg of protein per sample using total protein measured by the Piece BCA Protein Assay Kit (Thermo Fisher Cat. No. 23225).

TNF-α treatment

HUVECs were seeded into 35 mm dishes. Cells were treated with 10 ng/mL human TNF-α for 0, 1, 4, 8 or 24 h. CRNDE expression was quantified with qPCR.

CRNDE overexpression construct

The sequence of CRNDE, accession number NR_034105 (Supplementary Fig. 5c), was cloned into pUltra[1] to form pUltra-CRNDE. pUltra was a gift from Malcolm Moore (Addgene plasmid # 24129; http://n2t.net/addgene:24129 ; RRID:Addgene_24129). Primers are: forward 5'-GATGCTAGCGGGGTCTCGATCGCGCTATT-3' and reverse 5'-TTTTTTTGAACCTTTTTTTTTCGGATCCGTG-3'.

Supplementary Figure Legends

Supplementary Figure 1. mRNA distribution.

A, Principal component analysis of the RNA-seq data.

B, Chromosomal distribution of the differentially expressed mRNAs.

C, Heatmap of all differentially expressed mRNAs.

D, CRNDE knockdown reduces expression of ER stress related genes. HUVECs were treated with shNT or shCRNDE for 24 hours. The expression of ATF6, PERK, XBP1 and DDIT3 was examined by qPCR. *$p < 0.05$.

Supplementary Figure 2. Intergenic lncRNA distribution.

A, Chromosomal distribution of the differentially expressed lncRNAs.

B, Heatmap of all differentially expressed lncRNAs.

Supplementary Figure 3. Palmitic acid does not change CRNDE promoter activity.

A, HUVECs were transduced with the CRNDE promoter-bearing luciferase construct pGL3-CRNDE pro or pGL3, and promoter activity was measured by luciferase activity, with and without stress by palmitic acid. Data shown are representative of two independent experiments with similar trends. Values are mean ± SD of triplicates.

B, The sequence of cloned CRNDE promoter.

Supplementary Figure 4. TNF-α does not induce CRNDE.

HUVECs were treated with TNF-α for the indicated time points and CRNDE expression was measured by RT-qPCR. Data shown are mean ± SEM from three independent experiments.

Supplementary Figure 5. CRNDE shRNA effectively reduces CRNDE expression.

HUVECs were transduced with lentiviral shRNA targeting CRNDE and CRNDE expression was measured by RT-qPCR. Data shown are mean ± SEM from three independent experiments, *$p<0.01$.

Supplementary Figure 6. Full scans of p21 and GAPDH detection of representative blot found in Figure 5C.

Supplementary Figure 7. CRNDE knockdown in HAECs has a similar effect to CRNDE knockdown in HUVECs on endothelial cell migration and tube formation

A, HAECs were treated with the vector pUltra or pUltra-CRNDE for 24 h and then palmitic acid or vehicle control for 12 h before wounding. Images were taken from fixed cells 12 h after wounding. Representative images of 1 experiment is shown. Scale bars are 500 μm.

B, Quantification of (A) (*p<0.001). Data is mean ± SD from 1 experiment.

C, CRNDE knockdown potentiates the inhibitory effects of palmitic acid on tube formation. HAECs were treated as for wound healing before being seeded into Matrigel-coated wells. Images were taken from fixed wells after 3 h. Representative images of 1 experiment shown.

D, Quantification of tube formation in (C). Tube length, the number of segments, and the number of nodes. *p<0.05, **p<0.01, ***p<0.001. Data is mean ± SD from 1 experiment.

E, Quantification of tube formation in (C). Number of branches (*$p<1*10^{-5}$). Data is mean ± SD from one experiment.

Supplementary Figure 8. CRNDE lentiviral overexpression construct increases CRNDE expression.

HUVECs were transduced with pUltra containing CRNDE (pUltra-CRNDE) and CRNDE expression was measured by RT-qPCR

A, CRNDE expression. Data is mean ± SEM from two independent experiments *p<0.001.

B, Sequence of the CRNDE gene inserted.

Supplementary Figure 9. CRNDE overexpression has a similar effect to CRNDE knockdown on endothelial cell migration and tube formation

A, HUVECs were treated with the vector pUltra or pUltra-CRNDE for 24 h and then palmitic acid or vehicle control for 12 h before wounding. Images were taken from fixed cells 12 h after wounding. Representative images of 2 independent experiments were shown. Scale bars are 500 μm.

B, Quantification of (A) (*p<0.001). Data is mean ± SEM from 2 independent experiments.

C, CRNDE knockdown potentiates the inhibitory effects of palmitic acid on tube formation. HUVECs were treated as for wound healing before being seeded into Matrigel-coated wells. Images were taken from fixed wells after 3 h. Representative images of 2 independent experiments were shown.

D, Quantification of tube formation in (C). Tube length, the number of segments, and the number of nodes. *$p<0.05$, ***$p<0.01$, ****$p<0.001$. Data is mean ± SEM from 2 independent experiments.

E, Quantification of tube formation in (C). Number of branches (*$p<0.05$, ***$p<0.01$, ****$p<1*10^{-4}$). Data is mean ± SEM from 2 independent experiments.

References

1 Lou, E. *et al.* Tunneling nanotubes provide a unique conduit for intercellular transfer of cellular contents in human malignant pleural mesothelioma. *PloS one* **7**, e33093, doi:10.1371/journal.pone.0033093 (2012).

Supplementary Figure 1

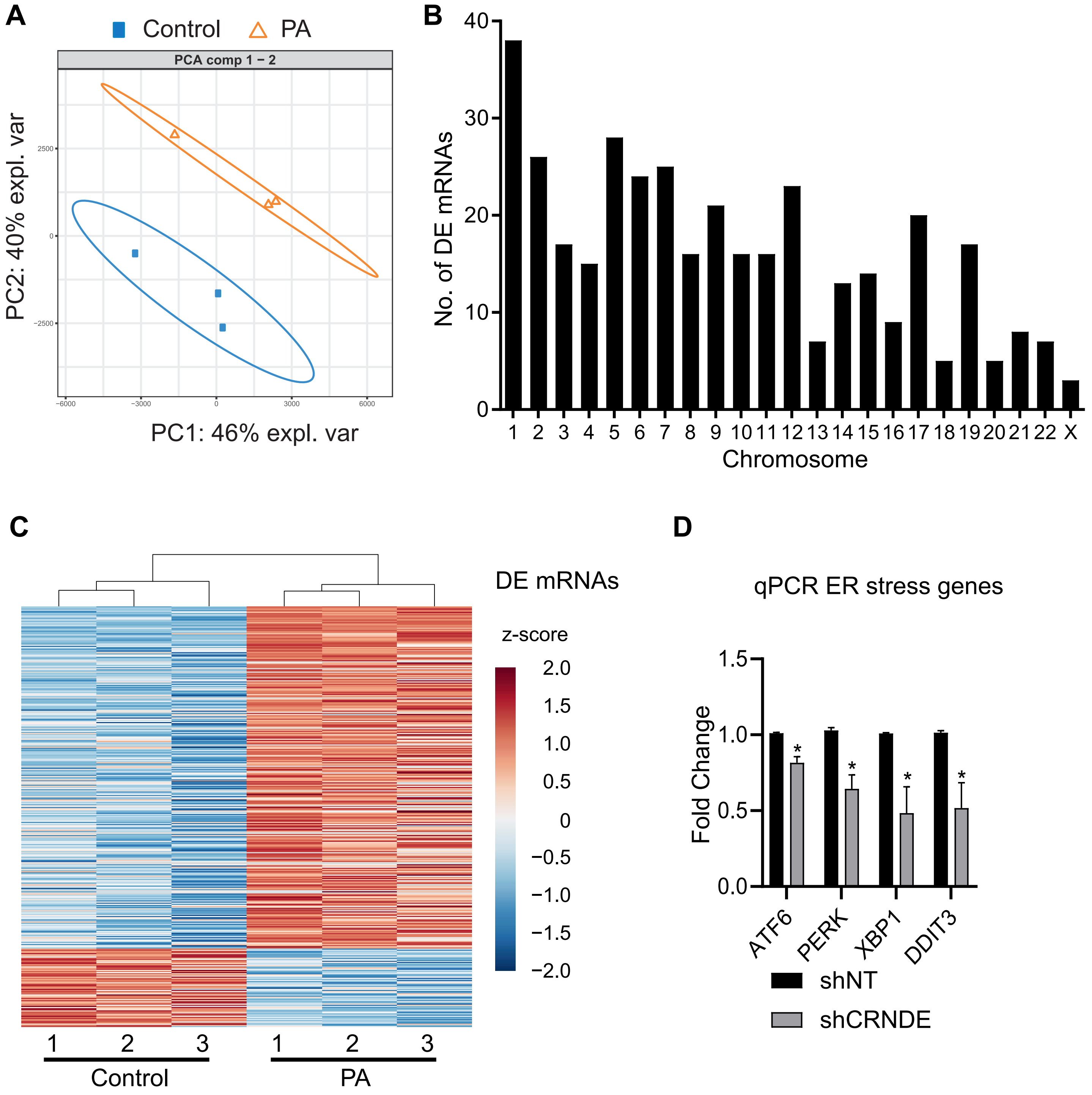

Supplementary Figure 2

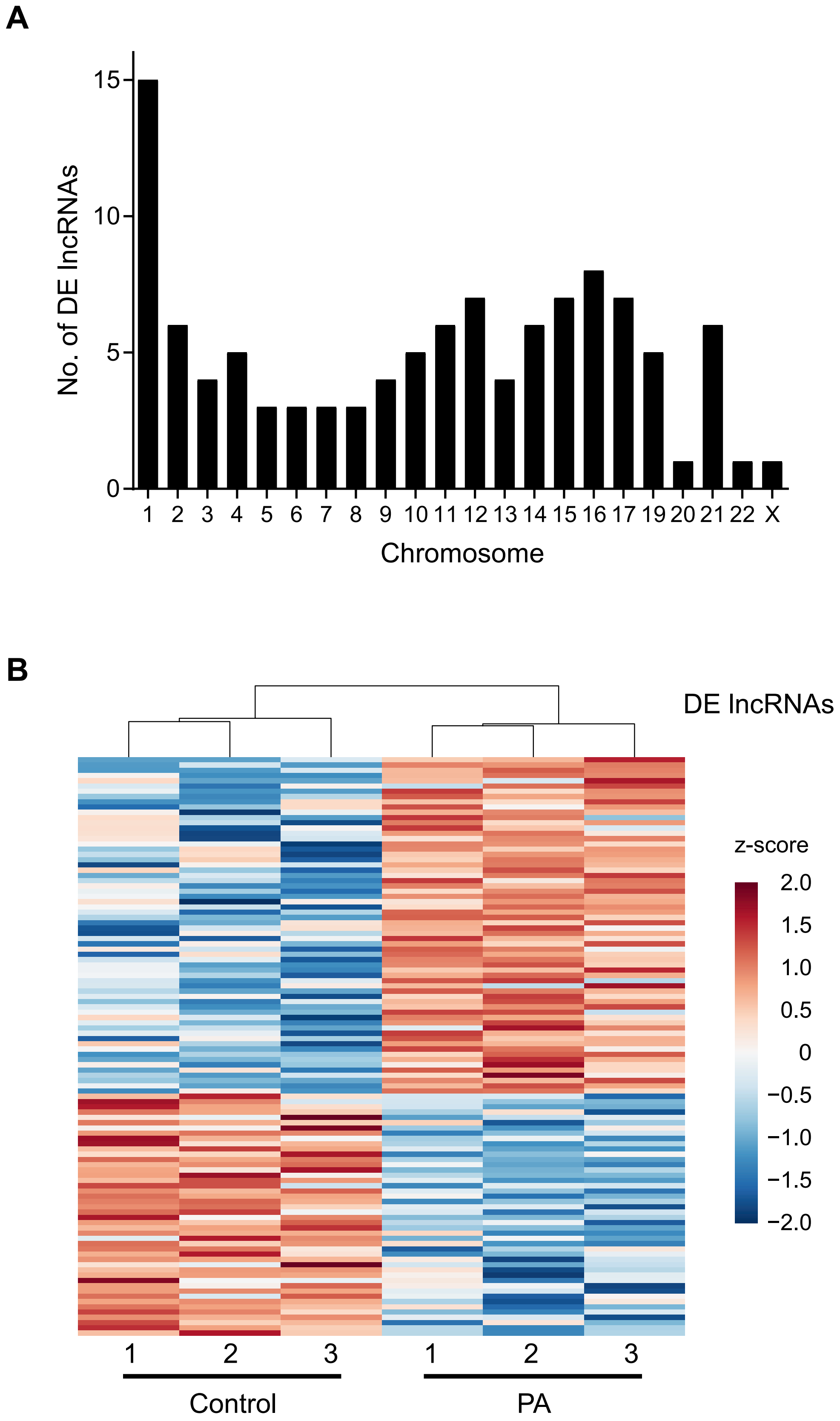

Supplementary Figure 3

A

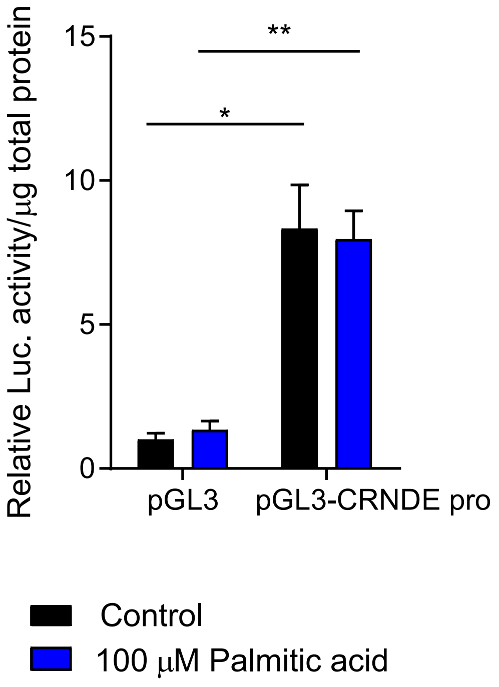

B

>CRNDE-promoter sequence
GGGGGCTCTGCTTTTGCCTTTGCAAAGGTCCTGCCAAGATGCTAAGTTGGAAATTGAGGATTCTGACGCCTTG
TGCGCGCCCGAAGCTCCTCCTTCCCGGGGTGTAGTCGGTGGGAGGACTGGCAGGAGCTTCTGGGCGGCCGCAG
CCAACCCGGCCGCCAGGCGCGCCTCGCCCTCTCCCTCTTCCTCCTCGGCTCTCTCTCCCGCTCGCTGGCGCTC
CTCTCGCCCCTCCCTAGCGCCCGCCTCCCCTCCGCGGCCCCCCCTCTCACCCCTCCTCTTTGCCTCCCCTTTT
CCCCTCCCCGGTCTCTCCCTCTCTCTCGCTTTCTCTCAGACTCTCGAGCGCCGGCCCCAGGATGACAATCACA
TCCCAGGAGCGCCGATCTCTTCCAACTTTCCTCTTCTGCTAACTCGGGGCGGAGTGGCAGTCCCGCCGCCCCG
AAGCACAAAGGGAACGAGGCCGCGGGCTGTGCGCCGGCGAACGCTCTGCGCTCTCCTAGCCACAGTAGATCGC
GGACTTAGCGGATTTCTTGTTCTCCGGCAGGCTGGGCTCCGAGGCCATTCGTTGCCCACCCCCCTCTGGCGTC
TTCCCCAAGCCAGGGGGCCCGGAGAGCCAGCTGGAGATCCGGAATGAAAGTCTCTGGGAGAGCCGATGGATGG
CCCGCGCCCAGGGCGCAGGAAGTCCGGGATGACTGCCCCTCTGCGCCGGCAGCAGCAGGTGGGCGAGAGACAG
GCCTCAGACGTCTGCACGTCTCCGCCTCGCCTTCCTTCTACCGACCCCCCGCCGGACGGCGAGGGAGAAGACA
CTGGTTCCTGGAACTACCGGGGTAGCCTTTTTCTAGAGTAGGGGTGGTGGGCAAGAACTGCCAGACAGAGAAT
CAGCTACACCCGAGGAGATCTCGGGACCGTCCCCAGCTCCACTCCCACGCCTCGCAGCCTCTTTCTCCCGGTT
TTCCCACCGCACGCAGCTCGCACCGCCAAGTCGGTGGTGGTGGAGGGTGCGGGTCCCCTTCCCTTTTGTTTAA
CTAAGTCGCCTTCCCCTTCGCACGCACTCTCGCATCCGCCCACGCTCCACTGCAAACACTGGGCAGAGCAGTG
CCCAACCCAACCACTGTTTGTTCAGCGAGGCGCTGGCGAAGCAAACACACACAACTGAGTCAGAGGCAGAGCC
CTTGCCCACGACGGCCACACAGTTAGAATACAAAACAGACCCTCTCCTCTTCTCTCCACCTCCCGCCACCACA
AGCCACGGACGCACTCGAACTCTGGGACAGACAGGGGCGCTGGTGAACCAGGACAGAAGATGGCACAGGTTTG
GGCACGCCGCGGAAGCTCGGGATATCGGGAACTTCGCATAATGGGGCCACACGCAAGCCGAAGCATAACTATA
TACAAAGTTTCTCGCATTGACTTTGACGGGCGAGATGTACTTTATTTCGCGATCTCACACTAACCCAGCGCCG
CACCGGCACCCGCTCGGTGCTGCGCTCTCGTGCACGCGCGTTGGCTCCTCCCCTCCGTCTGCTCCCCTCCCCC
AGACACCGCCCACCAAGAGGCCTGAGCGGTTCAGACTACATTCTCCGAGAGCCCCTGGGTCCGCCCAGCCCAG
TGCCTGACACCTCCTTCACCTATGATTGGGCGCTGGCCTCCCTGGGCTCCGCCCCCTGGTGACGTAACCCCGC
TTTCCTCCGAGTCTCGGCGCCAGAGGGGCGGGGAGGGGCGGGGTCTCGATCGCGCTATTGTCATGGAGACGGG
AAGCTGGCT

Supplementary Figure 4

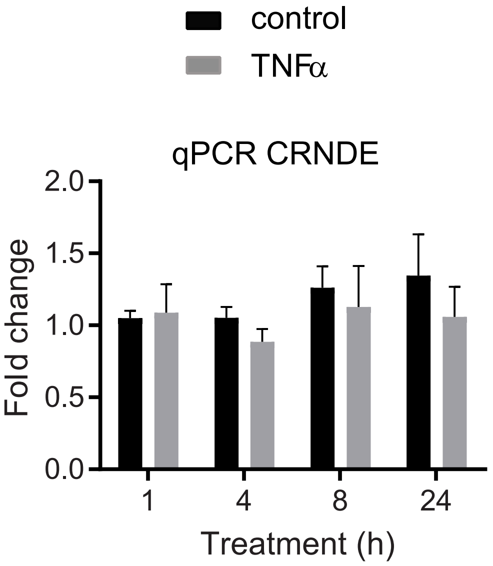

Supplementary Figure 5

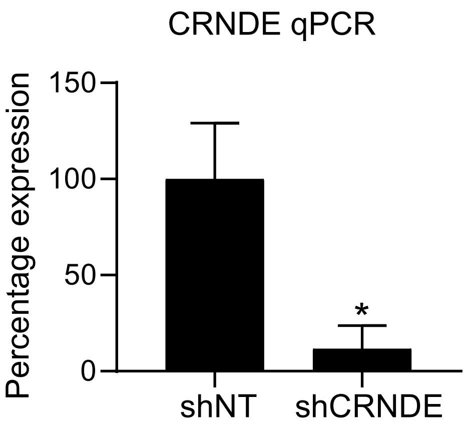

Supplementary Figure 6

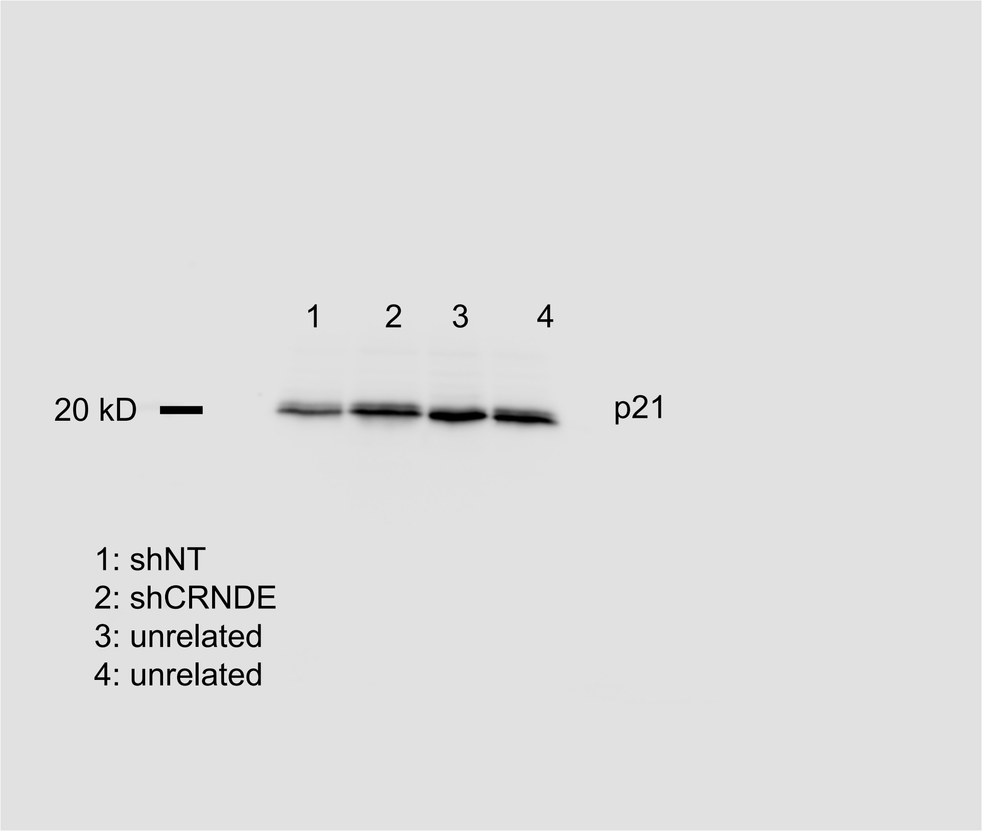

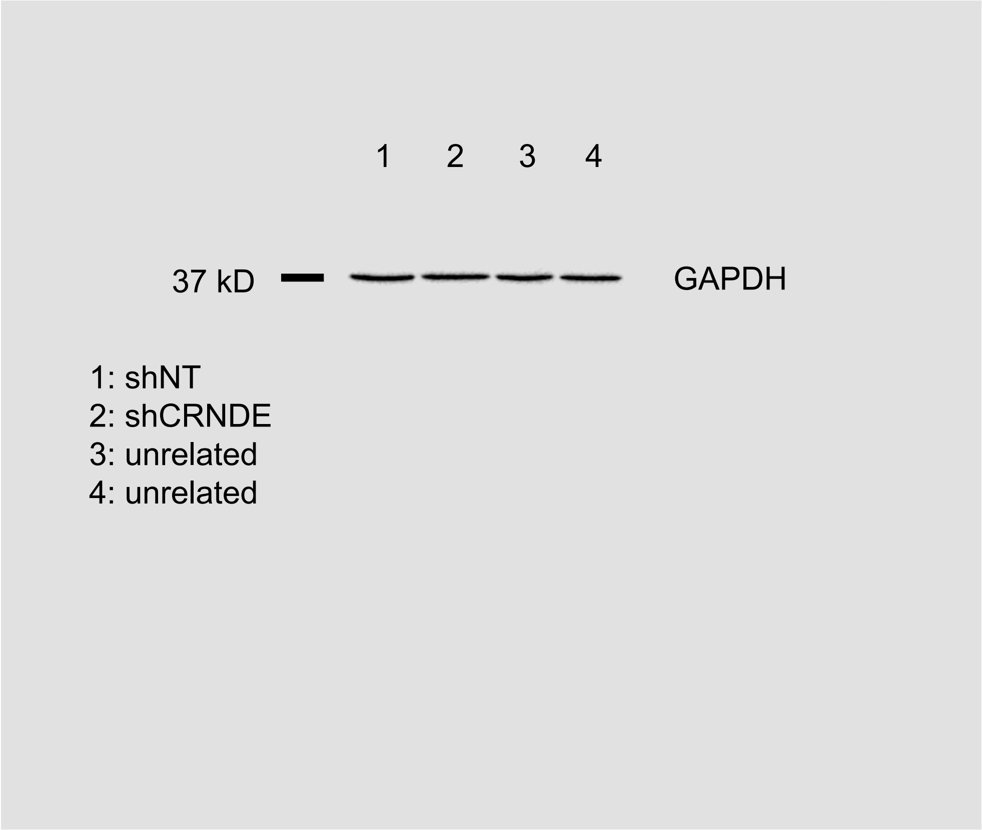

Supplementary Figure 7

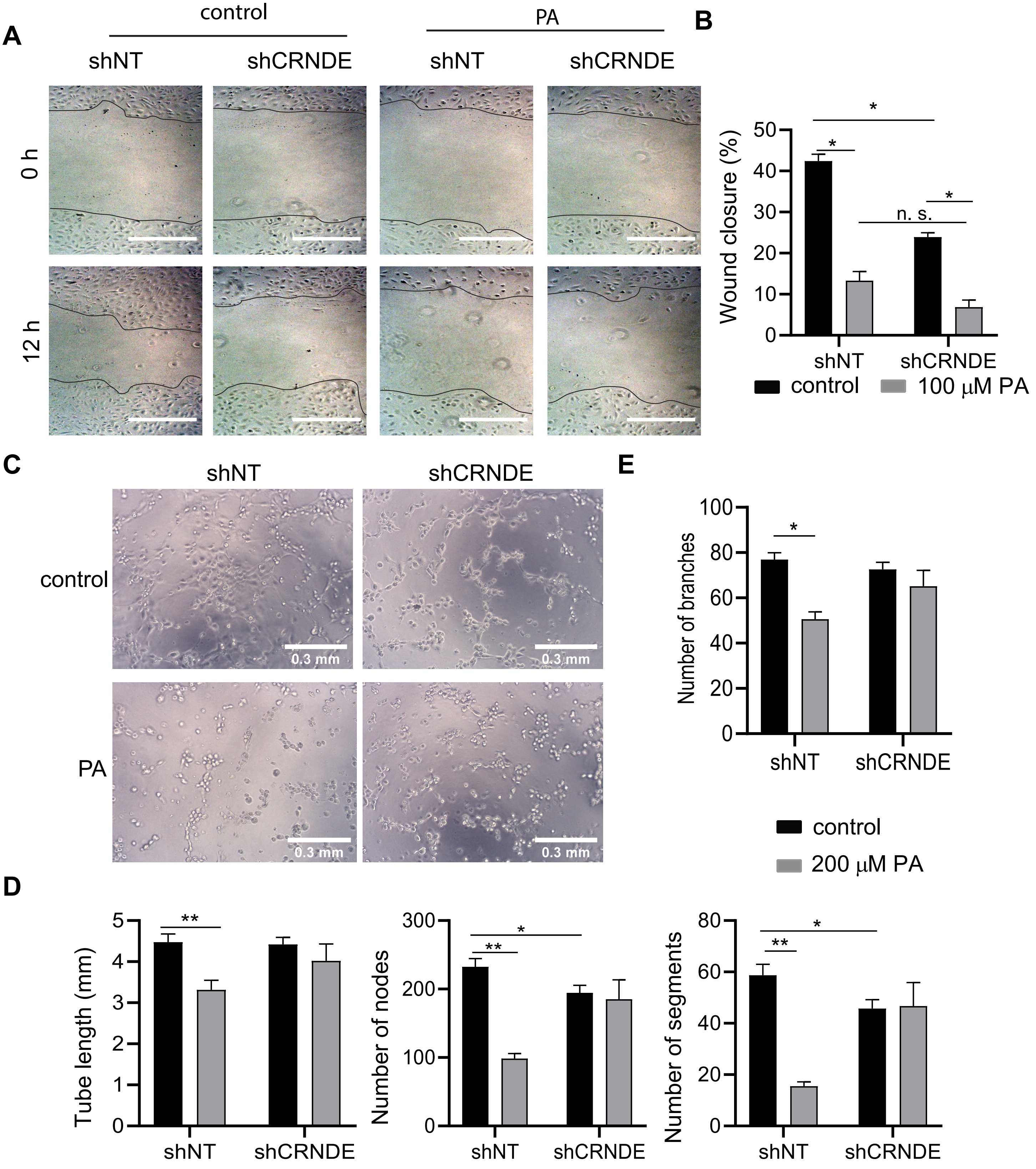

Supplementary Figure 8

A

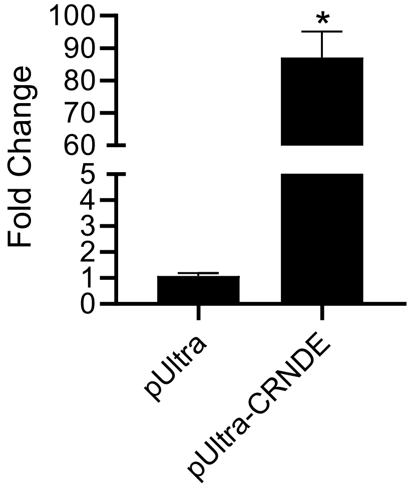

B

```
> CRNDE sequence
GGGGTCTCGATCGCGCTATTGTCATGGAGACGGGAAGCTGGCTGCAGCGGCGGCGGGGACCGTGGGGCCGAGGT
GGCTGCCAGCCGGCCAATGTCTAAGCGAGGCGGAGCGGCCCAGGCGGCCCGAGCCTGGGGGAGCGCGCAGCCGG
CCAGTGGCGGCCTCGCCGGCGGCCTCTTCCCGGGCTCGCAGTAGGCCCGAGTCGTCGCCGGGAGCTCCTGGGAG
CAGCGTCCCCGCCCTGCTCCCCTCGCTCCCGCCTCTTGCGGCCCCACGGCCCCTCAGCGCCCGCCCCCGGCTCC
GCCCGCCGCAGCCGCAGCCCCTGGCGCTAACGGTCGGTAACGGCCCGCGCGCGCCGCCCGCCGGGGGCTCGCGC
CAGCCACGAGGGAGCGTCCGCGGCCCGCGCGCCCGCGCGGCGGAGGAGAGGTGTTAAGTGTGATGCTTCCATAA
TACATTTGGATGCTGTCAGCTAAGTTCACTTCTGAACTAAGGGGTTCCTCCAAATGTTGGCTGAAATTCATCCC
AAGGCTGGTCTGCAAAGTCTGCAATTCATAATGGAGCTACTGTACTGGCTATTGGAAGGAGGAGATTCTGAAGA
TAAGGAGGTAAAACCTGTTTAGAAATTAAAAATGAGTTACGATTTAAAGAAAATTCAGATGACTCATTGTGAGT
GCTAGTTCTCTTGTAGGATGCCACTGGAAATGTTGAAATGAAAAATATTCAGCCGTTGGTCTTTGAAATTTCCT
GTGATGTGTTTCAATCTAGATGCAAAGAACATGGAAAAATCAAAGTGCTCGAGTGGTTTAAATATGTTTTGGGT
ATTCCTGTTTATAGACTATAATACTTTTCCAATTAAAATCCTCAGTTGTCACGCAGAAGAAGGTTAAGCTGTAT
TTGATTGCCAGTTTTACTGAAAATGCTTAGTATTTTACAGTATCACCAAATATATTTTGTTTAGCCAAGGTATA
GGAAAAATAAAATAAATTGTATAGGTTGACTTTTTTCTAAAATGTCTTTATTGGATTGAATGAATGTTTATACC
TGAAAAAAAAAGGTTCAAAAAAA
```

Supplementary Figure 9

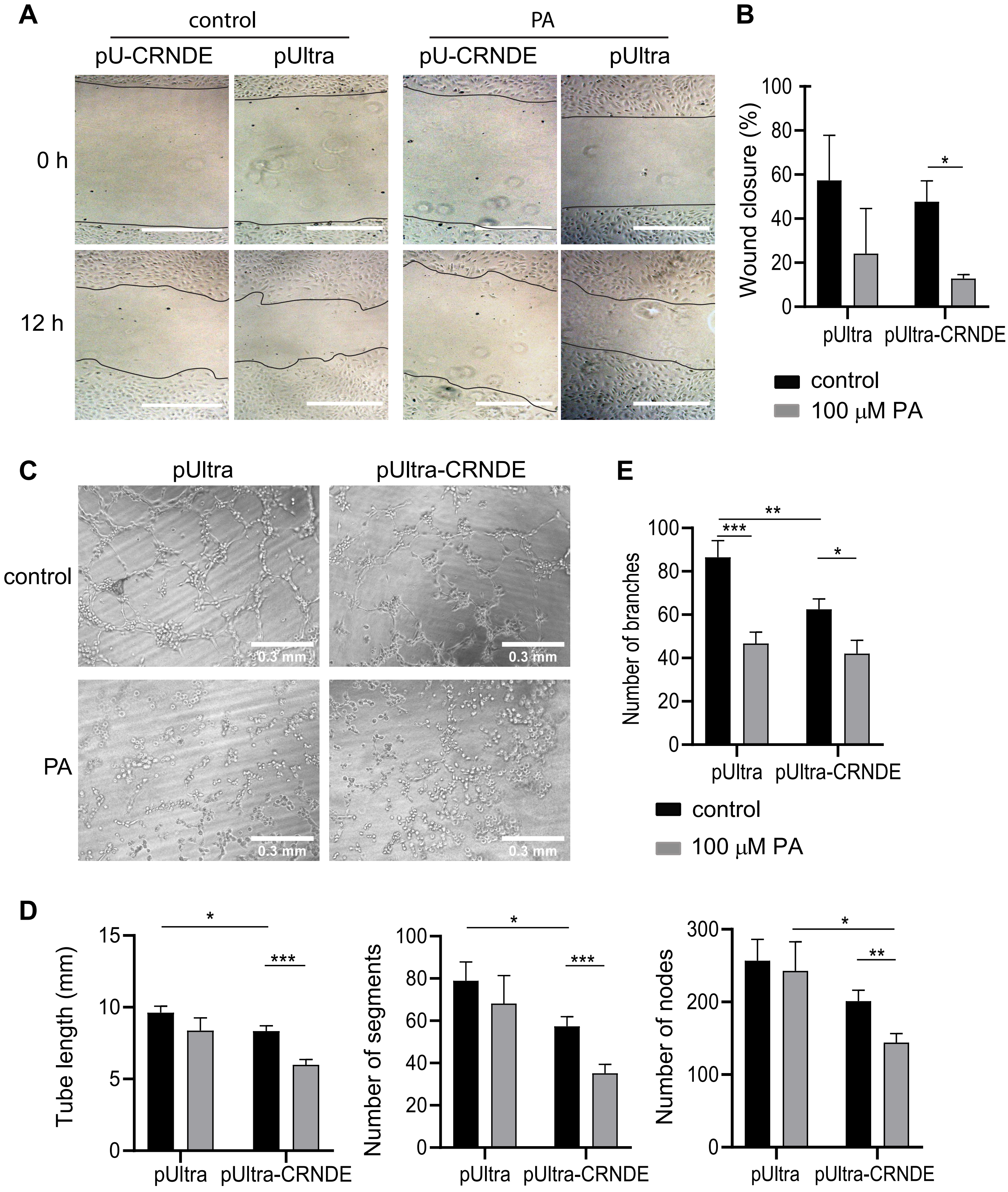

Supplementary Table 1. Alignment of reads to human genome by Tophat2.

Treatment	Replicate	Total Reads	Primary Aligned Reads	% Mapped Reads	% Paired Reads
Control	1	59,840,381	51,756,846	100.00 %	84.55 %
	2	67,122,243	57,707,780	100.00 %	82.84 %
	3	57,910,765	50,544,790	100.00 %	81.29 %
PA	1	80,309,248	68,965,663	100.00 %	82.66 %
	2	63,759,201	55,104,070	100.00 %	82.18 %
	3	79,760,115	69,144,774	100.00 %	81.78 %

Supplementary Table 2. CRNDE CPM values

Replicate	BSA	PA
1	3.02	5.23
2	3.17	5.18
3	2.70	4.21
Average Counts	224.0	608.67

Supplementary Table 3. Differentially Expressed lncRNAs.

Ensembl	Gene Symbol	logFC	PValue
ENST00000427169	RP11-495P10.5	4.15	1.95×10^{-3}
ENST00000530576	RP11-266A24.1	3.99	4.70×10^{-4}
ENST00000544086	RP11-253I19.3	3.90	1.10×10^{-3}
ENST00000566370	LINC00563	3.46	7.42×10^{-4}
ENST00000417975	RP11-475O6.1	3.29	2.57×10^{-2}
ENST00000434245	RP11-495P10.8	3.22	4.61×10^{-3}
ENST00000565305	RP11-1006G14.2	2.91	9.52×10^{-4}
ENST00000564531	RP11-1100L3.8	2.79	1.82×10^{-10}
ENST00000507248	RPL34-AS1	2.61	2.90×10^{-2}
ENST00000435368	AC023481.1	2.39	2.46×10^{-2}
ENST00000417096	AC007879.2	2.27	1.93×10^{-3}
ENST00000568695	RP11-92G12.3	2.26	2.94×10^{-2}
ENST00000568752	RP11-989E6.10	2.20	1.22×10^{-2}
ENST00000563464	RP11-1006G14.1	2.18	4.35×10^{-2}
ENST00000433770	RP11-472N13.3	2.18	1.44×10^{-2}
ENST00000442815	AP001046.5	2.13	2.11×10^{-2}
ENST00000612592	RP11-711K1.8	2.08	2.10×10^{-2}
ENST00000530725	LINC01301	2.03	2.28×10^{-2}
ENST00000441947	LINC00315	2.03	1.54×10^{-2}
ENST00000623723	CH507-39O4.2	2.01	2.38×10^{-2}
ENST00000611780	RP11-157L3.12	1.90	2.37×10^{-3}
ENST00000556370	RP3-449M8.6	1.87	1.93×10^{-2}
ENST00000606542	RP1-136B1.1	1.84	2.16×10^{-2}
ENST00000588501	LINC01483	1.80	4.26×10^{-3}
ENST00000573042	LINC01569	1.73	1.75×10^{-2}
ENST00000450760	RP11-211G3.2	1.68	1.66×10^{-2}
ENST00000366713	IBA57-AS1	1.67	2.83×10^{-2}
ENST00000444330	RP11-527D7.1	1.64	4.28×10^{-2}
ENST00000612269	CTD-2553L13.10	1.59	1.56×10^{-2}
ENST00000513492	LUCAT1	1.57	7.35×10^{-6}
ENST00000507639	RP11-328N19.1	1.52	2.40×10^{-5}

Ensembl	Gene Symbol	logFC	PValue
ENST00000558082	LINC01583	1.50	1.13×10^{-3}
ENST00000526605	RP11-347E10.1	1.48	2.94×10^{-2}
ENST00000421848	RP11-374M1.4	1.44	9.67×10^{-3}
ENST00000509098	RP11-663P9.1	1.40	3.00×10^{-2}
ENST00000541888	RP1-102E24.8	1.30	1.05×10^{-2}
ENST00000417335	AF064858.8	1.28	1.06×10^{-2}
ENST00000565506	RP11-354I13.2	1.25	1.30×10^{-2}
ENST00000570130	RP11-480A16.1	1.14	1.36×10^{-2}
ENST00000608792	LINC00702	1.12	8.24×10^{-5}
ENST00000608917	PACERR	1.12	4.81×10^{-3}
ENST00000454935	OLMALINC	1.01	6.68×10^{-4}
ENST00000607414	RP11-1017G21.5	1.00	4.38×10^{-2}
ENST00000562493	CTC-459F4.1	1.00	3.43×10^{-2}
ENST00000602927	RP11-646I6.5	0.97	2.57×10^{-2}
ENST00000609602	LINC00896	0.96	4.43×10^{-2}
ENST00000603468	RP11-359E10.1	0.96	1.44×10^{-3}
ENST00000454981	LINC01611	0.93	4.23×10^{-3}
ENST00000397841	LINC01547	0.86	3.15×10^{-2}
ENST00000605249	RP11-138A9.1	0.86	2.87×10^{-2}
ENST00000608412	RP11-138A9.2	0.83	9.05×10^{-4}
ENST00000542475	C17orf100	0.83	2.02×10^{-2}
ENST00000447748	LINC01359	0.81	2.72×10^{-2}
ENST00000452366	RP3-471M13.2	0.81	2.30×10^{-2}
ENST00000605834	RP11-326I11.3	0.78	3.74×10^{-2}
ENST00000623180	RP11-1228E12.1	0.75	1.65×10^{-3}
ENST00000501177	CRNDE	0.75	9.31×10^{-4}
ENST00000603682	RP1-142L7.9	0.72	5.61×10^{-4}
ENST00000466430	RP11-34P13.7	0.72	2.04×10^{-2}
ENST00000437712	RP11-168K11.3	0.72	2.62×10^{-2}
ENST00000567401	RP11-105C19.2	0.67	4.60×10^{-2}
ENST00000592431	CTD-2538C1.2	0.67	3.44×10^{-2}
ENST00000499986	AC138035.2	0.64	2.16×10^{-3}
ENST00000534991	URB1-AS1	0.61	3.86×10^{-2}
ENST00000566990	CTD-2515H24.4	−0.61	3.33×10^{-2}
ENST00000572818	RP11-160E2.11	−0.61	3.40×10^{-2}
ENST00000418344	RP5-1070A16.1	−0.67	3.31×10^{-3}
ENST00000613696	AC000403.4	−0.69	4.59×10^{-2}
ENST00000556652	CTD-2207P18.2	−0.72	4.86×10^{-2}
ENST00000569740	CTD-2015G9.2	−0.76	4.96×10^{-2}
ENST00000532350	MIR100HG	−0.78	4.21×10^{-2}
ENST00000429227	RP5-1061H20.4	−0.87	2.53×10^{-2}
ENST00000588040	CTD-2528L19.6	−0.91	3.45×10^{-2}
ENST00000443576	RP11-141M1.1	−0.92	2.34×10^{-2}
ENST00000596623	ZNF888	−0.93	3.24×10^{-3}
ENST00000623515	LINC01126	−0.98	2.62×10^{-2}
ENST00000443779	LINC00961	−0.99	6.98×10^{-6}
ENST00000415504	EWSAT1	−1.00	7.02×10^{-3}
ENST00000563734	RP11-304L19.13	−1.04	3.23×10^{-2}
ENST00000565617	KB-1460A1.5	−1.04	5.75×10^{-3}
ENST00000311067	RP11-89N17.1	−1.06	1.19×10^{-3}
ENST00000442355	RP11-160E2.17	−1.09	2.21×10^{-2}
ENST00000554829	RP11-356O9.1	−1.09	1.70×10^{-2}
ENST00000455974	AC123023.1	−1.09	4.45×10^{-2}
ENST00000588041	RP1-193H18.2	−1.14	9.49×10^{-6}
ENST00000355358	GATA3-AS1	−1.16	2.65×10^{-3}

Ensembl	Gene Symbol	logFC	PValue
ENST00000443092	SACS-AS1	−1.21	4.13×10^{-2}
ENST00000433133	AC078941.1	−1.23	2.48×10^{-2}
ENST00000436756	LINC01347	−1.25	2.94×10^{-2}
ENST00000558245	RP11-462P6.1	−1.30	8.30×10^{-3}
ENST00000549830	LINC00592	−1.35	2.01×10^{-2}
ENST00000608273	RP11-318E3.9	−1.36	2.34×10^{-2}
ENST00000557526	RP11-7F17.3	−1.43	1.39×10^{-2}
ENST00000574293	CTC-786C10.1	−1.50	3.48×10^{-2}
ENST00000503695	RP11-46H11.3	−1.58	1.63×10^{-2}
ENST00000529252	FAM66E	−1.59	5.50×10^{-3}
ENST00000452690	RP11-320G24.1	−1.62	3.07×10^{-2}
ENST00000602396	RP6-91H8.5	−1.63	2.04×10^{-2}
ENST00000445039	RP4-784A16.5	−1.81	3.82×10^{-2}
ENST00000533106	AP001257.1	−1.86	4.94×10^{-2}
ENST00000618746	RP11-370I10.11	−2.01	2.97×10^{-2}
ENST00000606089	CTB-113I20.2	−2.05	6.98×10^{-4}
ENST00000608316	RP11-367H1.1	−2.22	3.22×10^{-2}
ENST00000623351	RP5-1198O20.4	−2.31	2.64×10^{-3}
ENST00000565944	RP11-111J6.2	−2.33	1.24×10^{-2}
ENST00000591705	RP11-434D2.3	−2.36	4.76×10^{-5}
ENST00000428495	RP4-644L1.2	−2.58	4.77×10^{-3}
ENST00000631132	CH17-132F21.5	−2.75	8.41×10^{-3}
ENST00000531511	RP11-672A2.4	−3.21	8.91×10^{-3}
ENST00000568345	RP11-1007O24.2	−3.23	1.23×10^{-2}

Supplementary Table 4. Primer sequences for qPCR

Primer	Sequence
ATF6-f	TCGGTCAGTGGACTCTTAT
ATF6-r	CCAGTGACAGGCTTATCTTC
CRNDE-f	ACTGGCTATTGGAAGGAGGAGA
CRNDE-r	GGCATCCTACAAGAGAACTAGCAC
DDIT3-f	GGTATGAGGACCTGCAAGAGGT
DDIT3-r	CTTGTGACCTCTGCTGGTTCTG
HMOX1-f	ACTCCCTGGAGATGACTCCC
HMOX1-r	TCTTGCACTTTGTTGCTGGC
PERK-r	CCATACTACAAGAGGGAGAGGAACA
PERK-f	TGGGTTGTCGAGGAATCTGACT
VEGFA-f	TGCTGTGGACTTGAGTTGGGAG
VEGFA-r	CCTGGCCTTGCACATTCCTG
XBP1-f	CTGAGTCCGAATCAGGTGCAG
XBP1-f	ATCCATGGGGAGATGTTCTGG
